# Transferability and Fine Mapping of genome-wide associated loci for lipids in African Americans

**DOI:** 10.1186/1471-2350-13-88

**Published:** 2012-09-21

**Authors:** Adebowale Adeyemo, Amy R Bentley, Katherine G Meilleur, Ayo P Doumatey, Guanjie Chen, Jie Zhou, Daniel Shriner, Hanxia Huang, Alan Herbert, Norman P Gerry, Michael F Christman, Charles N Rotimi

**Affiliations:** 1 Center for Research on Genomics and Global Health, National Human Genome Research Institute, National Institutes of Health, Bethesda, MD, USA; 2 National Institute of Neurological Disorders and Stroke, National Institutes of Health, Bethesda, MD, USA; 3 Department of Genetics and Genomics, Boston University, Boston, MA, USA; 4 Coriell Institute for Medical Research, Camden, NJ, USA

**Keywords:** Lipids, Genetics, African Americans, Genome-wide association study, Ethnicity

## Abstract

**Background:**

A recent, large genome-wide association study (GWAS) of European ancestry individuals has identified multiple genetic variants influencing serum lipids. Studies of the transferability of these associations to African Americans remain few, an important limitation given interethnic differences in serum lipids and the disproportionate burden of lipid-associated metabolic diseases among African Americans.

**Methods:**

We attempted to evaluate the transferability of 95 lipid-associated loci recently identified in European ancestry individuals to 887 non-diabetic, unrelated African Americans from a population-based sample in the Washington, DC area. Additionally, we took advantage of the generally reduced linkage disequilibrium among African ancestry populations in comparison to European ancestry populations to fine-map replicated GWAS signals.

**Results:**

We successfully replicated reported associations for 10 loci (*CILP2/SF4*, *STARD3*, *LPL*, *CYP7A1*, *DOCK7*/*ANGPTL3*, *APOE*, *SORT1*, *IRS1*, *CETP*, and *UBASH3B*). Through trans-ethnic fine-mapping, we were able to reduce associated regions around 75% of the loci that replicated.

**Conclusions:**

Between this study and previous work in African Americans, 40 of the 95 loci reported in a large GWAS of European ancestry individuals also influence lipid levels in African Americans. While there is now evidence that the lipid-influencing role of a number of genetic variants is observed in both European and African ancestry populations, the still considerable lack of concordance highlights the importance of continued ancestry-specific studies to elucidate the genetic underpinnings of these traits.

## Background

The distribution of serum lipid concentrations has well-established clinical utility as a risk factor for a range of metabolic diseases, including cardiovascular disease (CVD) and type 2 diabetes (T2D). As such, great effort has gone into uncovering the genetic epidemiology of serum lipids, including a recent meta-analysis of 46 genome-wide studies comprising more than 100,000 individuals of European ancestry [1]. This study yielded 95 loci significantly associated with at least one of four serum lipid traits: high-density lipoprotein cholesterol (HDL), low-density lipoprotein cholesterol (LDL), triglycerides (TG), or total cholesterol (TC). Understanding the genetic underpinnings of risk factors for metabolic disorders is particularly relevant for African Americans, who experience a disproportionate burden of CVD mortality [2,3] and T2D [4]; this disparity is projected to continue [5]. While replication of some of the lipid-associated loci identified among European ancestry individuals has been reported for African Americans of the NHLBI’s CARe consortium [1,6] and the PAGE study [7], several limitations to these analyses warrant further evaluation. First, 20 of the lead associations in the 95 loci have not yet been investigated in African Americans (primarily due to the lack of availability of TC in these cohorts). Second, for all but a few associations that have been investigated, exact replication (*i.e.*, look-up of only the reported index SNP) was attempted. Given the generally greater linkage disequilibrium (LD) among European-ancestry individuals, it is expected that index SNPs tag larger regions than they would among African ancestry individuals. Therefore, the functional variant tagged by the index SNP in European ancestry individuals might not be in LD with the same SNP in African-ancestry individuals, motivating different analytical strategies (*i.e.*, “local” replication, described below). In the present study, we used more robust analytic strategies to investigate the transferability of reported genetic associations for serum lipids to African Americans. We also exploited these interethnic differences in LD to conduct fine-mapping of the replicated loci.

## Methods

Ethical approval for this study was obtained from the Howard University Internal Review Board. Written informed consent was provided by all participants. The Howard University Family Study (HUFS) is a population-based study of African Americans in the Washington, DC metropolitan area [8]. Unrelated, non-diabetic participants were included if they were not using lipid-lowering medication. Serum lipids were assayed using fasting blood samples (>8 hours), and concentrations were determined enzymatically on a Cobas Integra 400 Analyzer (Roche Diagnostics, Indianapolis, IN). The intra-assay coefficients of variation (CV) for lipid assays indicate consistent performance (TC, LDL, and HDL, CV <1.5%; TG, CV < 3.0%).

Genome-wide genotyping was performed using the Affymetrix® Genome-Wide Human SNP Array 6.0. Genotype calls were made using Birdseed, version 2 [9]. SNPs were excluded if they had a call rate <95% (n = 41,885), a minor allele frequency <0.01 (n = 19,154) or a Hardy-Weinberg equilibrium (HWE) test *p*-value <1 × 10^-3^ (n = 6,317). A total of 808,465 autosomal SNPs passed these filters. The average call rate for this set of SNPs was 99.55%, with concordance of blind duplicates of 99.74%. A check for population stratification was conducted using non-parametric clustering of genotypes as previously described [10].

Power calculations were performed using QUANTO [11]. When the MAF was at least 0.05, this study was adequately powered (power > 90%) to detect associations of the range observed in the prior publication for all traits except for HDL (power < 50% to detect the minimum effect size observed previously). With a MAF of 0.01, the power remained sufficient across the range of TG, but was low (≤50%) to detect the minimum effect sizes observed for each of the other traits. Imputation was performed using the MACH algorithm as previously described [8] (with 1000 Genomes reference data, http://www.1000genomes.org/). SNPs were excluded if they had a missingness rate ≥10%, Hardy-Weinberg test *p*-value <1 × 10^-3^, or a minor allele frequency <0.01. Imputed SNPs for which no rsid is currently available are described with a “chromosome:position” nomenclature (position refers to NCBI36 build). After quality control filters, 5,396,780 markers were included in the analysis.

Association analysis for the log-transformed lipid variables was performed using PLINK v1.07 [12] under an additive genetic model with adjustment for age, sex, body mass index (BMI), and the first 2 PCs of the genotypes (computed using EIGENSTRAT [13]). The appropriate number of PCs necessary to adjust for population substructure in HUFS has been previously determined [14]. Replication was attempted using two strategies. First, we investigated the exact SNPs that were previously identified [1]. A SNP was considered replicated if the direction of effect was consistent and the association *p*-value was ≤0.05 [15]. Second, we looked at all SNPs that were in LD with the reported SNPs in the CEU population, using a search window of ±250 kb from the index SNP with r^2^ ≥0.30 (“local replication”; for further discussion, see [16]). *P*-values obtained in the local replication were corrected for the effective degrees of freedom within an LD block containing the reported SNP [17], and an adjusted *p*-value 0.05 was considered statistically significant.

To take advantage of the generally decreased haplotype size in African ancestry populations, fine-mapping of replicated signals was attempted using the following strategies: inspection of regional plots of association to identify SNPs with a stronger signal than the index SNP in HUFS (LocusZoom 1.1, http://csg.sph.umich.edu/locuszoom/) and comparison of haplotype block structure between the CEU and YRI for SNPs of interest (Haploview 4.2, http://www.broadinstitute.org/haploview/haploview). Finally, we examined the other SNPs on the array and the imputed SNPs for any association with a genome-wide significant *p* < 2.5 × 10^-8^[18].

## Results

**Figure 1:**
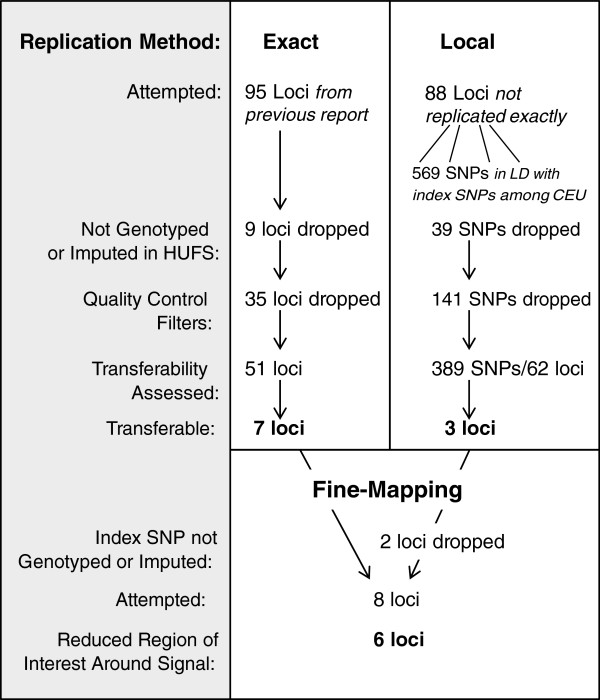
Summary of methods.

The study sample comprised 887 African Americans (374 men, 513 women), with a mean age of 46 years and a mean BMI of 28 kg/m^2^ in men and 31.5 kg/m^2^ in women (Table 1). Of the 95 previously identified lipids-associated index SNPs, 86 were successfully genotyped or imputed in HUFS (Figure 1). After quality control, 51 SNPs were included in the exact replication analysis. We successfully replicated 7 of these 51 previously identified lipids-associated loci: *CILP2/SF4*, *STARD3*, *LPL*, *CYP7A1*, *DOCK7/ANGPTL3*, *APOE,* and *SORT1* (Table 2). A comparison of the allele frequencies in those of African and European ancestry is provided for those SNPs that did not replicate (Additional file 1).

**Table 1:** Characteristics of study sample

**Characteristic**	**Men**	**Women**
N	374 (42%)	513 (58%)
Age (years)	45.5 ± 12.4	46.4 ± 13.3
Body mass index (kg/m^2^)	28.4 ± 7.3	31.5 ± 8.8
High density lipoprotein (mg/dL)	51.7 ± 17.5	54.8 ± 16.3
Low density lipoprotein (mg/dL)	113.6 ± 38.1	119.6 ± 38.7
Total cholesterol (mg/dL)	189.2 ± 41.4	196.8 ± 43.2
Triglycerides (mg/dL)	108.6 ± 61.4	100.1 ± 60.2

Data are number (percent) or mean ± standard deviation.

**Table 2:** **Exact replication of previously reported GWAS associations**^**1 **^**in a cohort of African Americans**

**SNP**	**Chr**	**Position**	**Minor Allele(Freq HUFS, Freq CEU)**	**Nearby Gene**	**Trait**	**HUFS**		**Previous Report**	
						**Beta (SE)**	***P*-value**	**Beta (SE)**	***P*-value**
rs10401969	19	19268718	G (0.17, 0.10)	*CILP2/SF4*	TG	−8.3 (2.8)	0.006	−7.8 (0.8)	1.6 x 10^-29^
rs11869286	17	35067382	G (0.26, 0.68)	*STARD3*	HDL	2.1 (0.9)	0.02	−0.5 (0.08)^2^	2.8 x 10^-14^
rs12678919	8	19888502	G (0.11, 0.12)	*LPL*	TG	−9.0 (4.4)	0.01	−13.6 (0.6)	1.5 x 10^-115^
rs2081687	8	59551119	T (0.22, 0.33)	*CYP7A1*	LDL	5.2 (2.5)	0.04	0.95 (0.2)	3.9 x 10^-9^
rs2131925	1	62798530	T (0.37, 0.68)	*ANGPTL3*	TC	4.5 (2.3)	0.04	−2.6 (0.2)^2^	4.9 x 10^-41^
rs4420638	19	50114786	C (0.21, 0.18)	*APOE*	HDL	−2.3 (0.9)	0.02	−1.1 (0.1)	4.4 x 10^-21^
rs629301	1	109619829	G (0.36, 0.30)	*SORT1*	LDL	−5.4 (1.9)	0.005	−5.7 (0.2)	9.7 x 10^-171^

^1^Teslovich *et al.*, Nature, 2010; ^2^The minor allele for HUFS is the major allele in previous report, thus the direction of effect should be reversed for comparison.

We replicated additional SNPs using an LD-based local replication strategy. We identified 569 SNPs that were in LD among the CEU with SNPs at the 88 loci that did not replicate exactly (Figure 1). Of these, 530 were genotyped or imputed in HUFS. After quality control, 389 SNPs representing 62 loci were included in the analysis. An additional 3 loci were replicated: *IRS1*, *CETP*, and *UBASH3B* (Table 3). In total, we were able to evaluate 82 of the 95 reported loci by either exact or local replication, and 10 of these (12%) showed significant association in HUFS.

**Table 3:** **Local replication of previously reported GWAS associations**^**1 **^**in a cohort of African Americans**

**Index SNP**	**Local SNP**	**R**^**2**^	**Chr**	**Position**	**Minor Allele (Freq HUFS, Freq CEU)**	**Nearby Gene**	**Trait**	**Local SNP Values)**	
								**Beta (SE)**	***P*-value**^**2**^
rs2972146	rs2943652	1	2	226816690	C (0.38, 0.40)	*IRS1*	HDL	3.0 (1.1)	0.01
rs3764261	rs173539	1	16	55545545	T (0.30, 0.37)	*CETP*	HDL	4.3 (1.2)	0.0003
	rs1800775	0.5	16	55552737	C (0.45, 0.49)	*CETP*	HDL	−2.5 (1.0)	0.03
	rs4783961	0.6	16	55552395	A (0.37, 0.51)	*CETP*	HDL	4.6 (1.3)	0.0009
rs7941030	rs6589939	0.9	11	122023735	G (0.35, 0.39)	*UBASH3B*	TC	−6.7 (2.1)	0.005

^1^Teslovich *et al.*, Nature, 2010; ^2^*P*-values have been adjusted for the effective degrees of freedom within the LD window surrounding the index SNP.

**Figure 2:**
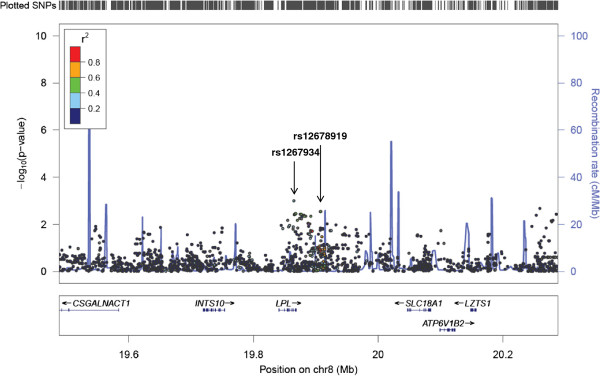
**Fine mapping of rs12678919. **LocusZoom output for the association of rs12678919 and TG, showing the association of nearby *LPL *SNP rs1267934, as well.

**Figure 3:**
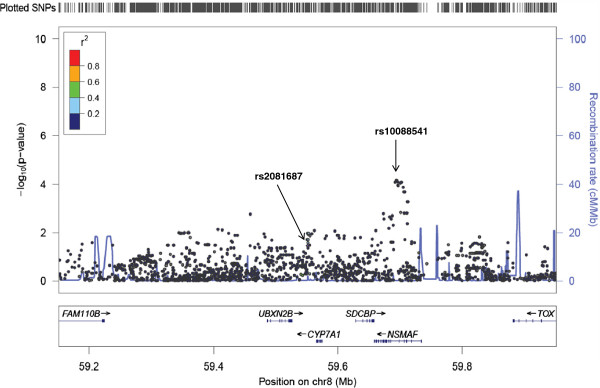
**Fine mapping of rs2081687. **LocusZoom output for the association of rs2081687 and LDL, showing the stronger association of nearby SNP rs10088541.

**Figure 4:**
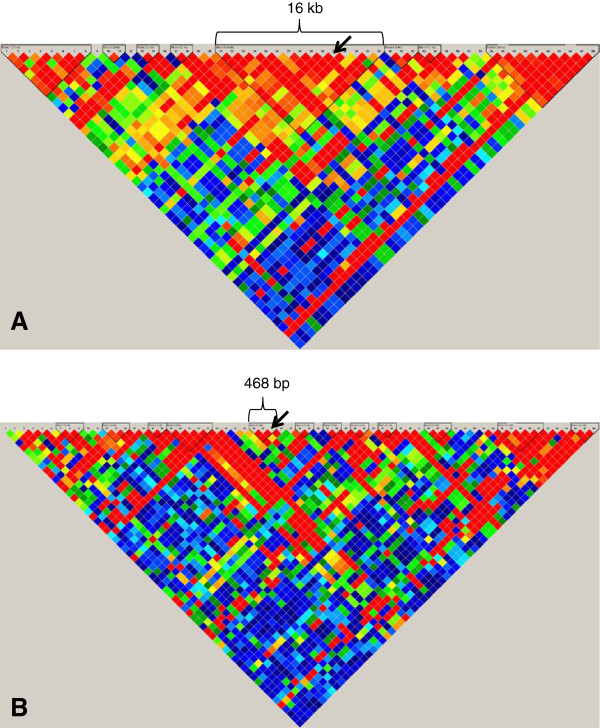
**Fine Mapping of rs629301. **Comparison of the haplotype structure in the region surrounding rs629301 (marked by an arrow) in the CEU (**A**) and the YRI (**B**).

For many of the 10 loci that were transferable, the generally reduced LD across the genomes of those of African ancestry resulted in finer mapping of the signals observed among European ancestry populations (Figure 1, Table 4). In the case of reported SNP rs12678919 (downstream of *LPL*), stronger association was observed for *LPL* intronic SNP rs12679834 (p = 0.001). While these two SNPs are in the same 53 kb haplotype block among the CEU, rs12678919 is not associated with a haplotype block in the YRI and rs12679834 is associated with a much smaller haplotype block (8 kb). This result suggests that the causal SNP is more closely linked with rs12679834 than rs12678919, and dramatically reduces the region for further investigation from 53 kb to 8 kb (Figure 2). Similarly, rs7941030 (*UBASH3B*) was not associated with TC in HUFS (p = 0.10), but rs6589939, which was in the same 40 kb haplotype block in the CEU, was (p = 0.005). Among the YRI, neither rs6589939 nor rs7941030 was in a haplotype block. In this study sample, an intronic SNP in *NSMAF*, rs10088541, had a lower p value than index SNP rs2081687 (nearest *CYP7A1*; p = 7.5 × 10–5 vs. 0.04). While these SNPs are correlated among the CEU (r^2^ = 0.75), they are not correlated in the HUFS samples (r^2^ = 0.03): the causal SNP may be more closely linked to rs10088541 (Figure 3). The replication of rs629301 (*SORT1*) in HUFS significantly reduces the region of interest for this signal. While this SNP is in a 16 kb haplotype block in the CEU, the block is reduced to less than 500 bp among the YRI (Figure 4).

**Table 4:** Summary of fine mapping of replicated loci

**SNP (local SNP if replicated locally)**	**Haplotype block length (CEU)**	**Haplotype block length (YRI)**	**Comment**
rs10401969	96 kb	88 kb	
rs11869286	19 kb	49 kb	
rs12678919	53 kb	Not in block	Higher peak is rs12679834 (p = 0.0010), in same haplotype block as rs12678919 in CEU, not in YRI. (Figure 2)
rs2081687	17 kb	1 kb	Rs10088541 is a higher peak nearby (p = 7.5 x 10^-5^). R^2^ between rs10088541 and index SNP = 0.75 among CEU, but only 0.03 in HUFS. (Figure 3)
rs2131925	298 kb	201 kb	
rs2972146 (rs2943652)			*Index SNP not successfully genotyped or imputed in HUFS: no fine-mapping comparisons possible.*
rs3764261 (rs173539)			*Index SNP not successfully genotyped or imputed in HUFS: no fine-mapping comparisons possible.*
rs3764261 (rs1800775)			*Index SNP not successfully genotyped or imputed in HUFS: no fine-mapping comparisons possible.*
rs3764261 (rs4783961)			*Index SNP not successfully genotyped or imputed in HUFS: no fine-mapping comparisons possible.*
rs4420638	Not in block	Not in block	
rs629301	16 kb	468 bp	(Figure 4)
rs7941030 (rs6589939)	40 kb	Not in block	Higher peak is at SNP rs6589939 (p = 0.005 vs. p = 0.10), which is in the same 40 kb block as the index SNP among CEU.

A search for other hits in the full set of genotyped and imputed SNPs showed that no SNPs reached genome-wide significance (Additional files 2, 3, 4, 5). The top SNPs were: chr16:50157331 near the gene *HEATR3* (associated with increased TG, *p* = 5.9 × 10^-8^), rs711794 near *ZAK* (associated with decreased LDL, *p* = 6.3 × 10^-8^), and rs1047163, a 3’ UTR variant near *HS1BP3* (associated with decreased HDL, *p* = 6.4 × 10^-8^).

## Discussion

We identified 10 loci that influence lipid levels in this cohort of African Americans. Of these, 7 were identified through testing the reported SNP while an additional 3 loci were identified using an LD-based strategy employed to account for the potential non-transfer of association signals across populations with different ancestral background [19]. Teslovich *et al.* assessed the generalizability of their findings by attempting replication in ~8,000 African Americans in the CARe consortium [1]. Of the 75 out of 95 loci for which the index SNP-trait association was investigated, 29 successfully replicated (see Supplementary Table 11 of that paper). A subset of these loci, along with replication of other lipid GWAS signals in the CARe African Americans, was also reported by Lettre *et al.*[6]). The PAGE study, which included ~9,000 African Americans, investigated 9 of 95 loci (all also included in CARe) and replicated 6 [7]. Of note, these were not independent samples, with both CARe and PAGE drawing participants from the ARIC and CARDIA cohorts. Of the 20 associations for which replication had not yet been attempted in an African American cohort, we were able to evaluate 16 in HUFS. One of these, an association between rs7941030 and TC, was replicated in HUFS. Additionally, four other associations that did not replicate in CARe were replicated in HUFS: rs10401969 (*CILP2/SF4*) with TG, rs2081687 (*CYP7A1*) with LDL, rs2972146 (*IRS1*) with HDL, and rs4420638 (*APOE*) with HDL (this association replicated in PAGE). CARe, PAGE, and HUFS all support the association of two loci with HDL in African Americans: rs3764261 (*CETP*) and rs4420638 (*APOE*).

Possible explanations for the lack of transferability of findings include differences in allele frequencies (see Additional file 1) and differences in effect sizes by population. Wide variability between populations in the frequency of risk alleles associated with a range of traits in GWAS has been demonstrated [20]. The correlation of effect sizes between GWAS-identified associations in European compared to African ancestry populations was only 0.27 (p = 0.2) in an evaluation of 24 SNPs with GWAS results for both ancestral groups. In fact, for 79% of the associations investigated, point estimates were in the opposite direction or differed by more than twofold in European vs. African ancestry comparisons [21]. Both of these results favor ancestry-specific analyses.

Some of the loci highlighted in this work have known biological functions relevant to serum lipids. STARD3, associated with HDL, is a lipid-trafficking protein. LPL, associated with TG, is a triglyceride hydrolase and a ligand factor for receptor-mediated lipoprotein uptake; mutations causing LPL deficiency have been implicated in type I hyperlipoproteinemia (NCBI: *LPL*, 2011). ApoE, associated with HDL, is a main lipoprotein of the chylomicron and is involved in the catabolism of triglyceride-rich lipoprotein constituents; defects in the gene encoding this protein result in familial dysbetalipoproteinemia (NCBI: *APOE*, 2011). CETP, associated with HDL, plays multiple roles in HDL metabolism and in the reverse cholesterol transport pathway [22]. A *CETP* SNP (rs247617) that was unlinked with the replicated SNP was one of the top hits for HDL in our discovery GWAS (Additional file 3), suggesting the presence of multiple functional variants at this locus. Based on searches of both the GWAS catalog [23] and PubMed, only one of the top SNPs from our discovery GWAS had been previously reported: rs247617, a variant 5 KB upstream of *CETP*, was also associated with HDL among Finns [24] and African Americans of the CARe consortium [6], with a consistent direction of effect. This variant appears to be a significant determinant of HDL concentration across ethnicities.

Our study has two main strengths and one main limitation. First, HUFS represents the general population of African Americans in the Washington, DC area. The lack of selection for disease status makes this an optimal study sample for drawing conclusions regarding transferability to a broader population of African Americans. Second, a local replication strategy was employed to evaluate transferability of the reported associations, in recognition of the well-known differences in LD structure across the genome between African and European ancestry individuals. The main limitation of this study is the modest sample size. In some instances, it is probable that the failure to replicate was a result of lack of power. For instance, rs9987289 (*PPP1R3B*) – HDL, which was replicated in the CARe consortium analysis, was not genotyped or imputed in this sample, but a local SNP, rs6601299 (r^2^ = 0.86) was associated in the same direction, but just above the significance level (*p* = 0.07). As the previous publication is a meta-analysis with a very large sample size, it was able to detect small effect sizes, which would be difficult to replicate in a GWAS with a more limited sample size. As a result, more accurate estimates of transferability will await the aggregation of African ancestry GWAS into a suitably large meta-analysis. Of the 10 replicated loci in this study, only 1 had been previously identified in an individual GWAS (rs3764261 and HDL in GWAS of Indian Asian men [25,26], Finns [27], and Japanese [28]).

## Conclusions

Overall, this study conducted in African Americans, replicated 10 of the 95 loci that were identified in a large GWAS of lipids in European ancestry populations. Together with results from previous work, there is now support for the transferability of 42% (40/95) of the European ancestry-identified loci to African Americans. Notably, conclusive inferences about the transferability of all of the previous findings are precluded by the limitations in replication attempts conducted in African Americans thus far in terms of relative sample size and coverage of African ancestry genetic diversity by currently available GWAS chips. Further work in African ancestry populations will be necessary to completely evaluate these loci.

## Competing interests

The authors declare that they have no competing interests.

## Authors’ contributions

AA and ARB analyzed the data, prepared figures and tables, and drafted the manuscript. KGM also conducted analyses. APD and HH assayed serum lipids. GC prepared the genome-wide genotype data. GC, DS, and AA conducted the genome-wide imputation of genotypes. AH, NPG, and MFC genotyped the samples. CNR, AA, ARB, and KGM contributed to the conception and design of the study. All authors read, provided important feedback, and approved the final manuscript.

## Pre-publication history

The pre-publication history for this paper can be accessed here:

http://www.biomedcentral.com/1471-2350/13/88/prepub

## Supplementary Material

Additional file 1 Allele Frequency Comparison for Tested Loci that were not Transferable. Click here for file

Additional file 2 Top SNPs for GWAS of Total Cholesterol. Click here for file

Additional file 3 Top SNPs for GWAS of HDL. Click here for file

Additional file 4 Top SNPs for GWAS of LDL. Click here for file

Additional file 5 Top SNPs for GWAS of TG. Click here for file
