# Supplementary material for: Transferability and Fine Mapping of genome-wide associated loci for lipids in African Americans
Source: BMC Med Genet. 2012 Sep 21;13:88. doi: 10.1186/1471-2350-13-88 (PMC3573912; doi:10.1186/1471-2350-13-88)
Supplement: Additional file 1 — Allele Frequency Comparison for Tested Loci that were not Transferable. [file 1471-2350-13-88-S1.pdf]

Additional File 1. Allele Frequency Comparison for Tested Loci that were not Transferable

SNP	Chr	Risk/Reference Alleles[1]	*Risk Allele Frequency* European Ancestry[2]	African Americans[3]
Comparable Allele Frequencies (n=20)				
rs1689800	1	G/A	0.35	0.32
rs2479409	1	G/A	0.3	0.27
rs645040	3	G/T	0.22	0.30
rs442177	4	G/T	0.41	0.47
rs6450176	5	A/G	0.26	0.30
rs1564348	6	C/T	0.17	0.11
rs3177928	6	A/G	0.16	0.08
rs17145738	7	T/C	0.12	0.09
rs10761731	10	T/A	0.43	0.39
rs3136441	11	C/T	0.15	0.21
rs964184	11	G/C	0.13	0.19
rs4759375	12	T/C	0.06	0.09
rs4765127	12	T/G	0.34	0.35
rs2412710	15	A/G	0.02	0.06
rs2652834	15	A/G	0.2	0.29
rs16942887	16	A/G	0.12	0.20
rs12967135	18	A/G	0.23	0.29
rs492602	19	G/A	0.49	0.49
rs2277862	20	T/C	0.15	0.17
Frequency considerably higher in AA[4] (n=12)				
rs4846914	1	G/A	0.4	0.86
rs10195252	2	C/T	0.4	0.74
rs6882076	5	T/C	0.35	0.63
rs2814944	6	A/G	0.16	0.32
rs605066	6	C/T	0.42	0.56
rs11776767	8	C/G	0.37	0.67
rs1495741	8	G/A	0.22	0.38
rs10128711	11	T/C	0.28	0.8
rs2923084	11	G/A	0.17	0.48
rs838880	12	C/T	0.31	0.68
rs2929282	15	T/A	0.05	0.33
rs6029526	20	A/T	0.47	0.79
Frequency considerably lower in AA[4](n=12)				
rs2642442	1	C/T	0.32	0.21
rs4660293	1	G/A	0.23	0.05
rs7515577	1	C/A	0.21	0.06
rs12916	5	C/T	0.39	0.25

rs4731702	7	T/C	0.48	0.28
rs2293889	8	T/G	0.41	0.13
rs174546	11	T/C	0.34	0.09
rs11613352	12	T/C	0.23	0.11
rs1169288	12	C/A	0.33	0.07
rs7134594	12	C/T	0.47	0.26
rs11649653	16	G/C	0.4	0.08
rs7255436	19	C/A	0.47	0.29

[1] As listed in the European Ancestry Meta-analysis

[2] From the European Ancestry Meta-Analysis

[3] From HUFS. The frequency of the previously-reported risk allele is given so that frequency columns are directly comparable.

[4] Variants where interethnic differences in allele frequency exceeded 10%.
