# Supplementary material for: Transferability and Fine Mapping of genome-wide associated loci for lipids in African Americans
Source: BMC Med Genet. 2012 Sep 21;13:88. doi: 10.1186/1471-2350-13-88 (PMC3573912; doi:10.1186/1471-2350-13-88)
Supplement: Additional file 2 — Top SNPs for GWAS of Total Cholesterol. [file 1471-2350-13-88-S2.pdf]

Additional File 2. Top SNPs for GWAS of Total Cholesterol

SNP	*P*-value	Beta (95% CI)	Minor Allele	MAF	Chr	Position	Nearest Gene	SNP Type	Detection Method[1]
chr10:63290442	9.11E-07	-34.3 (-46.1, -21.5)	A	0.02	10	63290442	*ARID5B*	Upstream	I
chr20:25533926	2.02E-06	-14.7 (-20.3, -8.8)	G	0.11	20	25533926	*NANP*	Upstream	I
chr10:63300324	2.06E-06	-38 (-51.4, -23.4)	T	0.01	10	63300324	*ARID5B*	Upstream	I
rs57830607	2.13E-06	-14.7 (-20.3, -8.8)	T	0.11	20	25515370	*NANP*	Downstream	I
chr8:72797070	2.20E-06	-25.3 (-34.7, -15.3)	G	0.03	8	72797070	*MSC*	Upstream	I
rs4075687	2.32E-06	10 (5.8, 14.3)	A	0.41	5	30505930	*RP11-136H13.2*	Intergenic	I
rs920279	2.37E-06	18.3 (10.6, 26.4)	G	0.09	2	127628659	*AC110926.2*	Intergenic	I
chr20:25502711	2.43E-06	-14.4 (-20, -8.6)	A	0.11	20	25502711	*KIAA0980*	Intronic	I
rs58516525	2.43E-06	-14.4 (-20, -8.6)	A	0.11	20	25505294	*KIAA0980*	Intronic	I
chr20:25509161	2.43E-06	-14.4 (-20, -8.6)	A	0.11	20	25509161	*KIAA0980*	Intronic	I
rs10271961	2.49E-06	-14.1 (-19.7, -8.4)	G	0.14	7	56261590	*CCNJP1*	Downstream	I
rs4867270	2.68E-06	9.9 (5.7, 14.2)	C	0.38	5	30487171	*RP11-136H13.2*	Intergenic	G
chr20:25532854	3.12E-06	-15.4 (-21.5, -9.1)	T	0.09	20	25532854	*NANP*	Upstream	I
rs6867293	3.13E-06	10 (5.7, 14.3)	T	0.44	5	30504885	*RP11-136H13.2*	Intergenic	I
chr20:25563200	3.37E-06	-14.1 (-19.7, -8.3)	A	0.11	20	25563200	*NANP*	Downstream	I
rs7500836	3.88E-06	11.5 (6.6, 16.5)	C	0.22	16	85509652	*AC106745.1*	Intergenic	I
chr20:25547693	3.90E-06	-15.2 (-21.2, -8.9)	A	0.09	20	25547693	*NANP*	Intronic	I
rs6450780	4.34E-06	9.6 (5.5, 13.8)	A	0.45	5	30503459	*RP11-136H13.2*	Intergenic	I
rs13345276	4.39E-06	36.7 (20.3, 54.2)	T	0.02	19	39251381	*RPS4XP21*	Intergenic	I
rs4797274	4.42E-06	-25.2 (-34.9, -15)	C	0.03	18	6969102	*LAMA1*	Intronic	I

[1]Whether SNP was genotyped (G) or imputed (I)
