# Supplementary material for: Transferability and Fine Mapping of genome-wide associated loci for lipids in African Americans
Source: BMC Med Genet. 2012 Sep 21;13:88. doi: 10.1186/1471-2350-13-88 (PMC3573912; doi:10.1186/1471-2350-13-88)
Supplement: Additional file 3 — Top SNPs for GWAS of HDL. [file 1471-2350-13-88-S3.pdf]

Additional File 3. Top SNPs for GWAS of HDL

SNP	*P*-value	Beta (95% CI)	Minor Allele	MAF	Chr	Position	Nearest Gene	SNP Type	Detection Method[1]
rs1047163	6.44E-08	-7 (-9.3, -4.6)	C	0.08	2	20681293	*HS1BP3*	Intronic	G
rs1897433	3.52E-07	4.8 (2.9, 6.7)	A	0.24	2	134032992	*NCKAP5*	Intronic	G
rs1195239	3.93E-07	6.2 (3.8, 8.8)	T	0.13	7	68311814	*RP11-3P22.2*	Intergenic	I
rs57444374	4.38E-07	-6.8 (-9.2, -4.3)	C	0.08	2	20699144	*HS1BP3*	Intronic	I
rs61227046	4.38E-07	-6.8 (-9.2, -4.3)	G	0.08	2	20700819	*HS1BP3*	Intronic	I
rs72782390	4.41E-07	-6.9 (-9.3, -4.4)	A	0.08	2	20684949	*HS1BP3*	Intronic	I
rs57347707	4.41E-07	-6.8 (-9.2, -4.3)	T	0.08	2	20700945	*HS1BP3*	Intronic	I
rs60580455	4.41E-07	-6.8 (-9.2, -4.3)	A	0.08	2	20702228	*HS1BP3*	Intronic	I
rs57417650	4.41E-07	-6.8 (-9.2, -4.3)	T	0.08	2	20704597	*HS1BP3*	Intronic	I
rs10194140	5.26E-07	4.8 (2.9, 6.8)	T	0.22	2	134049571	*NCKAP5*	Intergenic	G
rs1897434	5.45E-07	4.8 (2.9, 6.8)	T	0.22	2	134033073	*NCKAP5*	Intronic	I
rs11675147	1.02E-06	5.3 (3.1, 7.6)	A	0.19	2	134043638	*NCKAP5*	Upstream	I
rs10197126	1.06E-06	-7.4 (-10.1, -4.6)	T	0.06	2	29367455	*ALK*	Intronic	I
rs13412991	1.18E-06	4.7 (2.8, 6.7)	C	0.22	2	134039507	*NCKAP5*	Intronic	I
rs978097	1.34E-06	4.7 (2.8, 6.7)	A	0.22	2	134044181	*NCKAP5*	Upstream	I
chr2:134041755	1.42E-06	4.7 (2.8, 6.7)	A	0.22	2	134041755	*AC010974.1*	Downstream	I
rs1307512	1.59E-06	-5 (-6.9, -3)	T	0.15	1	91085272	*RP4-665J23.1*	Intronic	G
rs13000827	1.85E-06	4.6 (2.7, 6.6)	C	0.24	2	134058287	*AC010974.1*	Intergenic	I
rs6587981	2.28E-06	4.6 (2.7, 6.6)	G	0.24	1	48878916	*AGBL4*	Intronic	I
rs4945933	2.29E-06	-4.1 (-5.7, -2.4)	G	0.24	6	113646886	*RP1-236J16.1*	Upstream	G
rs4439062	2.45E-06	6.5 (3.7, 9.4)	C	0.10	7	125277824	*AC005276.1*	Intergenic	I
rs4439063	2.45E-06	6.5 (3.7, 9.4)	T	0.10	7	125278107	*AC005276.1*	Intergenic	I
chr11:31974761	3.02E-06	-7.5 (-10.3, -4.5)	T	0.06	11	31974761	*RCN1*	Intergenic	I
rs247617	3.15E-06	4.3 (2.5, 6.2)	A	0.26	16	55548217	*CETP*	Intergenic	G
chr7:148245243	3.38E-06	-14 (-18.7, -8.6)	A	0.01	7	148245243	*EZH2*	Downstream	I
rs7549812	3.62E-06	4.5 (2.6, 6.5)	T	0.24	1	48876505	*AGBL4*	Intronic	I
rs6708174	4.01E-06	4.6 (2.6, 6.7)	C	0.22	2	134060713	*AC010974.1*	Intergenic	I
rs1188926	4.14E-06	-5 (-6.9, -2.9)	A	0.14	1	91088864	*ZNF644*	Intergenic	I
chr20:6508845	4.38E-06	-12.8 (-17.3, -7.8)	G	0.02	20	6508845	*BMP2*	Intergenic	I
rs4793515	4.78E-06	-4 (-5.6, -2.3)	A	0.29	17	66462101	*AC005181.1*	Intergenic	I
rs7754776	4.83E-06	6.4 (3.6, 9.3)	A	0.09	6	13373877	*PHACTR1*	Inronic	G

[1]Whether SNP was genotyped (G) or imputed (I)
