# Supplementary material for: Transferability and Fine Mapping of genome-wide associated loci for lipids in African Americans
Source: BMC Med Genet. 2012 Sep 21;13:88. doi: 10.1186/1471-2350-13-88 (PMC3573912; doi:10.1186/1471-2350-13-88)
Supplement: Additional file 4 — Top SNPs for GWAS of LDL. [file 1471-2350-13-88-S4.pdf]

Additional File 4. Top SNPs for GWAS of LDL

SNP	*P*-value	Beta (95% CI)	Minor Allele	MAF	Chr	Position	Nearest Gene	SNP Type	Detection Method[1]
rs711794	6.28E-08	-12.2 (-16.2, -8)	C	0.21	2	173854697	*ZAK*	Downstream	I
rs820044	6.84E-08	-11.9 (-15.9, -7.8)	A	0.21	2	173853469	*ZAK*	Downstream	I
rs820042	6.84E-08	-11.9 (-15.9, -7.8)	T	0.21	2	173853723	*ZAK*	Downstream	G
rs4797274	2.63E-07	-25.8 (-33.9, -16.8)	C	0.03	18	6969102	*LAMA1*	Intronic	I
rs73126777	5.61E-07	-33.3 (-43.6, -21.7)	T	0.01	4	31505789	*AC097654.2*	Intergenic	I
rs10991534	5.71E-07	-21.9 (-29.3, -13.9)	A	0.03	9	106892035	*SLC44A1*	Intergenic	G
chr18:24285215	7.11E-07	-37.7 (-48.9, -24.6)	G	0.01	18	24285215	*CDH2*	Intergenic	I
chr5:126615349	7.15E-07	-37.6 (-48.8, -24.6)	G	0.01	5	126615349	*MEGF10*	Upstream	I
rs7379343	8.19E-07	-26.3 (-35, -16.7)	T	0.02	5	84372188	*AC113412.1*	Intergenic	G
rs6768039	1.05E-06	-26.4 (-35.2, -16.7)	T	0.02	3	27387602	*SLC4A7*	Downstream	I
chr10:63290442	1.18E-06	-31.9 (-42, -20.3)	A	0.02	10	63290442	*ARID5B*	Upstream	I
rs315677	1.39E-06	12.5 (7.3, 17.9)	G	0.20	4	24990879	*ANAPC4*	Intronic	G
rs647296	1.46E-06	-23.8 (-32, -14.8)	A	0.03	3	174641219	*NLGN1*	Intronic	I
chr10:63300324	1.46E-06	-35 (-46, -22.3)	T	0.013	10	63300324	*ARID5B*	Upstream	I
chr9:18916807	1.47E-06	-31.9 (-42.2, -20.2)	G	0.02	9	18916807	*FAM154A*	Upstream	I
chr16:78351295	1.72E-06	18.3 (10.6, 26.6)	C	0.08	16	78351295	*MAF*	Intergenic	I
rs10830976	1.78E-06	-10.5 (-14.5, -6.4)	C	0.24	11	92452436	*RPL26P31*	Intergenic	I
rs41311218	2.42E-06	-23 (-31.1, -14.1)	G	0.03	9	137850661	*CAMSAP1*	Intronic	I
chr11:18991104	3.32E-06	-33.3 (-44.3, -20.7)	A	0.01	11	18991104	*MRGPRX2*	Upstream	I
chr11:18991837	3.32E-06	-33.3 (-44.3, -20.7)	A	0.01	11	18991837	*MRGPRX2*	Upstream	I
chr11:18993141	3.32E-06	-33.3 (-44.3, -20.7)	T	0.01	11	18993141	*MRGPRX2*	Upstream	I
rs16936007	3.32E-06	-33.3 (-44.3, -20.7)	A	0.01	11	18997152	*MRGPRX2*	Upstream	I
rs16936009	3.32E-06	-33.3 (-44.3, -20.7)	A	0.01	11	18997970	*MRGPRX2*	Upstream	I
chr11:18999650	3.32E-06	-33.3 (-44.3, -20.7)	A	0.01	11	18999650	*MRGPRX2*	Upstream	I
chr11:18984380	3.47E-06	-33.3 (-44.2, -20.7)	A	0.01	11	18984380	*MRGPRX2*	Upstream	I
chr5:126600724	3.71E-06	-32.4 (-43.2, -20)	T	0.02	5	126600724	*MEGF10*	Intergenic	I
chr4:31521821	3.98E-06	-31.7 (-42.3, -19.5)	C	0.01	4	31521821	*AC094654.2*	Intergenic	I
rs13406521	4.05E-06	-16.5 (-22.7, -9.8)	T	0.07	2	20980013	*AC012361.1*	Intergenic	G
rs17116062	4.06E-06	-23.5 (-31.9, -14.2)	T	0.03	5	153754313	*GALNT10*	Intronic	I
rs10271961	4.40E-06	-12.9 (-18, -7.6)	G	0.14	7	56261590	*CCNJP1*	Downstream	I
rs1383551	4.76E-06	-9 (-12.6, -5.2)	C	0.40	15	98433423	*ADAMTS17*	Intronic	I
rs8180784	4.78E-06	-11.3 (-15.8, -6.7)	A	0.18	7	56248594	*CCNJP1*	Intergenic	I

[1]Whether SNP was genotyped (G) or imputed (I)
