# Supplementary material for: Transferability and Fine Mapping of genome-wide associated loci for lipids in African Americans
Source: BMC Med Genet. 2012 Sep 21;13:88. doi: 10.1186/1471-2350-13-88 (PMC3573912; doi:10.1186/1471-2350-13-88)
Supplement: Additional file 5 — Top SNPs for GWAS of TG. [file 1471-2350-13-88-S5.pdf]

Additional File 5. Top SNPs for GWAS of TG

SNP	*P*-value	Beta (95% CI)	Minor Allele	MAF	Chr	Position	Nearest Gene	SNP Type	Detection Method[1]
chr16:50157331	5.90E-08	63.1 (37, 94)	G	0.02	16	50157331	*AC084058.6-1*	Intergenic	I
chr20:56173824	3.60E-07	36.7 (21.3, 53.8)	T	0.03	20	56173824	*C20orf85*	Downstream	I
chr16:50154664	3.91E-07	46.6 (26.7, 69.6)	T	0.03	16	50154664	*AC084058.6-1*	Intergenic	I
chr20:56171067	6.03E-07	35.2 (20.3, 52)	T	0.03	20	56171067	*C20orf85*	Downstream	I
chr20:56171725	6.03E-07	35.2 (20.3, 52)	A	0.03	20	56171725	*C20orf85*	Downstream	I
chr20:56171885	6.03E-07	35.2 (20.3, 52)	T	0.03	20	56171885	*C20orf85*	Downstream	I
chr20:56172094	6.03E-07	35.2 (20.3, 52)	C	0.03	20	56172094	*C20orf85*	Downstream	I
chr20:56173287	6.03E-07	35.2 (20.3, 52)	T	0.03	20	56173287	*C20orf85*	Downstream	I
chr16:2881419	1.08E-06	76.2 (40.7, 120.4)	A	0.01	16	2881419	*FLYWCH1*	Intronic	I
chr16:2904218	1.55E-06	81.3 (42.7, 130.2)	A	0.01	16	2904218	*FLYWCH1*	Intronic	I
rs4750962	1.67E-06	20.8 (11.9, 30.3)	A	0.10	10	130166324	*Rp11-264E18.1*	Intergenic	I
chr16:50292828	1.69E-06	70.6 (37.5, 111.7)	A	0.01	16	50292828	*AC084058.6-1*	Downstream	I
rs9765940	1.81E-06	44.1 (24.3, 67.2)	C	0.02	6	150132817	*PCMT1*	Intronic	I
chr16:2900872	2.02E-06	76.8 (40.2, 122.9)	G	0.01	16	2900872	*FLYWCH1*	Upstream	I
chr16:2901743	2.02E-06	76.8 (40.2, 122.9)	T	0.01	16	2901743	*FLYWCH1*	Upstream	I
chr16:2891098	2.08E-06	64.8 (34.4, 102)	T	0.02	16	2891098	*FLYWCH1*	Upstream	I
chr16:2891329	2.08E-06	64.8 (34.4, 102)	C	0.02	16	2891329	*FLYWCH1*	Upstream	I
chr16:2904226	2.54E-06	82.8 (42.6, 134.1)	G	0.01	16	2904226	*FLYWCH1*	Intronic	I
chr1:167219749	2.87E-06	62.7 (33, 98.8)	G	0.01	1	167219749	*ATP1B1*	Intergenic	I
chr16:50173931	2.89E-06	41.9 (22.7, 63.9)	C	0.03	16	50173931	*AC084058.6-1*	Intergenic	I

[1]Whether SNP was genotyped (G) or imputed (I)
